# Deep Learning in Mining Biological Data

**DOI:** 10.1007/s12559-020-09773-x

**Published:** 2021-01-05

**Authors:** Mufti Mahmud, M. Shamim Kaiser, T. Martin McGinnity, Amir Hussain

**Affiliations:** 1 Department of Computer Science, Nottingham Trent University, Clifton, NG11 8NS Nottingham, UK; 2 Institute of Information Technology, Jahangirnagar University, Savar 1342 Dhaka, Bangladesh; 3 Intelligent Systems Research Centre, Ulster University, Northern Ireland BT48 7JL Derry, UK; 4 School of Computing , Edinburgh, EH11 4BN Edinburgh, UK; 5 Medical Technology Innovation Facility, Nottingham Trent University, NG11 8NS Clifton, Nottingham, UK

**Keywords:** Brain–Machine Interfaces, Bioimaging, Deep learning performance comparison, Medical imaging, Omics, Open access data sources, Open-source tools

## Abstract

Recent technological advancements in data acquisition tools allowed life scientists to acquire multimodal data from different biological application domains. Categorized in three broad types (i.e. images, signals, and sequences), these data are huge in amount and complex in nature. Mining such enormous amount of data for pattern recognition is a big challenge and requires sophisticated data-intensive machine learning techniques. Artificial neural network-based learning systems are well known for their pattern recognition capabilities, and lately their deep architectures—known as deep learning (DL)—have been successfully applied to solve many complex pattern recognition problems. To investigate how DL—especially its different architectures—has contributed and been utilized in the mining of biological data pertaining to those three types, a meta-analysis has been performed and the resulting resources have been critically analysed. Focusing on the use of DL to analyse patterns in data from diverse biological domains, this work investigates different DL architectures’ applications to these data. This is followed by an exploration of available open access data sources pertaining to the three data types along with popular open-source DL tools applicable to these data. Also, comparative investigations of these tools from qualitative, quantitative, and benchmarking perspectives are provided. Finally, some open research challenges in using DL to mine biological data are outlined and a number of possible future perspectives are put forward.

## Introduction

The pursuit of understanding human behaviours, along with the various pathologies, their early diagnosis and finding cures, has driven the life sciences research in the last two centuries [1]. This accelerated the development of cutting edge tools and technologies that allow scientists to study holistically the biological systems as well as dig down, in an unprecedented manner, to the molecular details of the living organisms [2, 3]. Increasing technological sophistication has presented scientists with novel tools for DNA sequencing [4], gene expression [5], bioimaging [6], neuroimaging [7], and body/brain–machine interfaces [8].

**Fig. 1:**
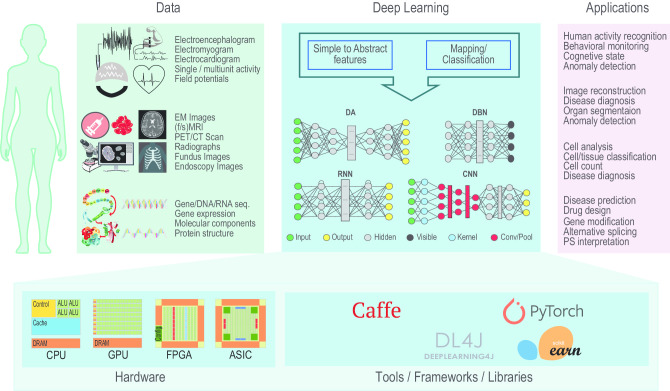
The ecosystem of modern data analytics using advanced machine learning methods with specific focus on application of DL to biological data mining. The biological data coming from various sources (e.g. sequence data from the *Omics*, various images from the *[Medical/Bio]-Imaging*, and signals from the *[Brain/Body]–Machine Interfaces*) are mined using DL with suitable architectures tailored for specific applications

These innovative approaches to study living organisms produce huge amount of data [9] and create a situation often referred as ‘Data Deluge’ [10]. Depending on the target application and experimentation, these biological big data can be characterized by their inherent characteristics of being *hierarchical* (i.e. data coming from different levels of a biological system—from molecules to cells to tissues to systems), *heterogeneous* (i.e. data acquired by different acquisition methods—from genetics to physiology to pathology to imaging), *dynamic* (i.e. data changes as a function of time), and *complex* (i.e. data describing nonlinear biological processes) [11]. These intrinsic characteristics of biological big data posed an enormous challenge to data scientists to identify patterns and analyse them to infer meaningful conclusions from these data [12]. The challenges have triggered the development of rational, reliable, reusable, rigorous, and robust software tools [11] using machine learning (ML)-based methods to facilitate recognition, classification, and prediction of patterns in the biological big data [13].

Based on how a method learns from the data, the ML techniques can be broadly categorized into *supervised* and *unsupervised* approaches. In *supervised* learning, objects in a pool are classified using a set of known annotations or attributes or features, i.e. a *supervised* algorithm learns the pattern(s) from a limited number of annotated training data and then classifies the remaining testing data using the acquired knowledge. Instead, in the *unsupervised* learning, pattern(s) are first defined from a subset of the unknown data and then the remaining data are classified based on the defined patterns, i.e. an *unsupervised* algorithm first defines pattern(s) among the objects in a pool of data with unknown annotations or attributes or features, and then uses the acquired knowledge to classify the remaining data. In addition, there is another category called *reinforcement* learning which is out of the scope of this work, but allows an agent to improve its experience and knowledge by learning iteratively through interacting with its environment.

Since the 1950s, many methods pertaining to both the learning paradigms (i.e. *supervised* and *unsupervised*) have been proposed. The popular methods in the *supervised* domain include: ANN [14] and its variants (e.g. Backpropagation [15], Hopfield Networks [16], Boltzmann Machines [17], Restricted Boltzmann Machines [18], Spiking Neural Networks [19], etc.), Bayesian Statistics [20], Support Vector Machines [21] and other linear classifiers [22] (e.g. Fisher’s Linear Discriminant [23], Regressors [24], Naive Bayes Classifier [25], etc.), k-Nearest Neighbours [26], Hidden Markov Model [27], and Decision Trees [28]. Popular *unsupervised* methods include: Autoencoders [29], Expectation–Maximization [30], Information Bottleneck [31], Self-Organizing Maps [32], Association Rules [33], Hierarchical Clustering [34], k-Means [35], Fuzzy Clustering [36], and Density-based Clustering [37, 38] (e.g. Ordering Points To Identify the Clustering Structure [39]). Many of these methods have been successfully applied to data coming from various biological sources.

For the sake of simplicity, the vast amount of biological data coming from the diverse application domains have been categorized to a few broad data types. These data types include *Sequences* (data generated by Omics technologies, e.g. [gen/transcript/epigen/prote/metabol]omics [40]), *Images* (data generated by [bio/medical/clinical/health] imaging techniques containing [sub-]cellular and diagnostic images), and *Signals* (electrical signals generated by the brain and the muscles and acquired using appropriate sensors at the [Brain/Body]–Machine Interfaces or BMI). Each of these data types originating at diverse biological application domains have witnessed major contributions from the specified ML methods and their variants (see for *Sequences* [41], *images* [42–44], and *signals* [45–47]).

**Fig. 2:**
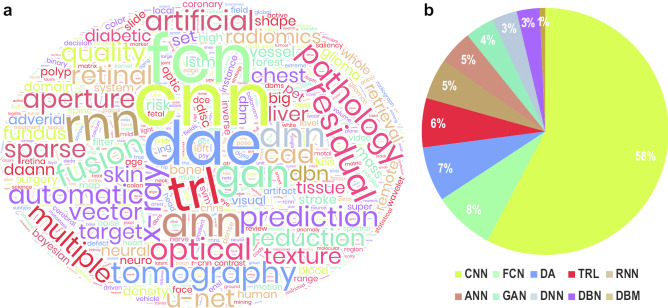
Application of different DL models to biological data. **a** Wordcloud generated using author keywords extracted from research papers published between January 2011 and March 2020 which mentioned analysis of biological data (images, signals and sequences) using DL techniques and indexed in the Scopus database. The keywords were pruned to highlight the analysis methods. **b** Distribution of published papers mentioning the usage of top 10 techniques. The colours of the individual pies match the colours in the wordcloud. Legend—CNN: Convolutional Neural Network, FCN: Fully Connected Network, DA[E]: Deep Autoencoder, TRL: Transfer Learning, RNN: Recurrent Neural Network (including Long Short-Term Memory or LSTM), ANN: Artificial Neural Network, GAN: Generative Adversarial Network, DNN: Deep Neural Network, DBN: Deep Belief Network, DBM: Deep Boltzmann Machine

In recent years, DL methods are potentially reshaping the future of ML and AI [48]. It is worthy to mention here that, from a broader perspective, ML has been applied to a range of tasks including anomaly detection [49, 50, 278, 283, 290], biological data mining [51, 52], detection of coronavirus [53, 54], disease detection and patient management [55–57, 277, 279–282, 284, 286, 287, 289, 291], education [58], natural language processing [59, 285, 288], and price prediction [60]. Despite notable popularity and applicability to diverse disciplines [61], there exists no comprehensive review which focuses on pattern recognition in biological data and provides pointers to the various biological data sources and DL tools, and the performances of those tools [51].

Also, considering the ecosystem of modern data analysis using advanced ML techniques (such as DL), providing information about methods application only partially covers the components of this ecosystem (see the various components of the ecosystem in Fig. 1). The remaining components of the ecosystem include open access data sources and open-source toolboxes and libraries which are used in developing the individual methods. It is therefore of paramount importance to have a complete understanding of the availability of datasets and their characteristics, the capabilities and options offered by the libraries, and how they compare with each other in different execution environments such as central processing unit (CPU) and graphical processing unit (GPU). The current paper’s novelty lies in being first of its kind to cover comprehensively the complete ecosystem of modern data analysis using advanced ML technique, i.e., DL.

Therefore, with the above aim, this review provides—a brief overview on DL concepts and their applications to various biological data types; a list of available open access data repositories offering data for method development; and a list of existing open-source libraries and frameworks which can be utilized to harness the power of these techniques along with their relative and performance comparison. Towards the end, some open issues are identified and some speculative future perspectives are outlined.

The remainder of the article is organized as follows: Section 2 provides the conceptual overview and introduces the reader to the underlying theory of DL; Section 3 describes the applications; Section 4 lists the open-source data repositories; Section 5 presents the popular open-source DL tools; and Sections 6 and 7 compare the most popular tools from relative and performance perspectives. Section 8 presents the reader with some of the open issues and hints on the future perspectives, and finally, the article is concluded in Section 9.

**Table 1:** Keypoints and applications of different deep learning architectures

Architecture	Pros.	Cons.
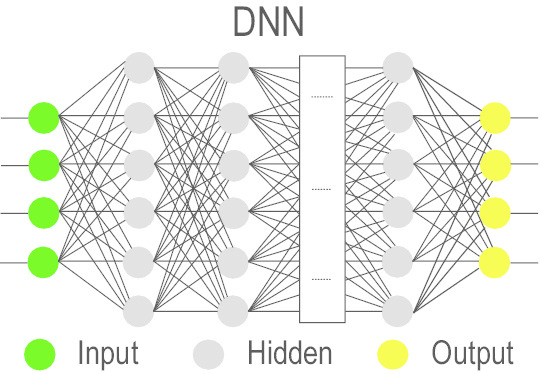	- DNN can learn high-level feature representation and apply transfer learning. - It can be used for healthcare and visual recognition.	- It requires substantial volume of training data. - Significant computational power is required. - The learning process is slow.
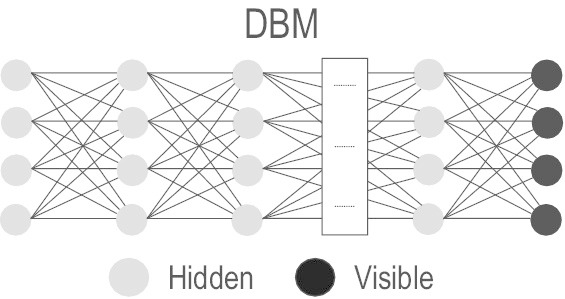	- Graphical model, undirected links across a set of visible nodes and a set of hidden nodes. - Used mainly for dimensionality reduction and classification.	- High time complexity for interference than DBN. ↵ - Learning information does not reach to the lower layer. - Tends to overfit.
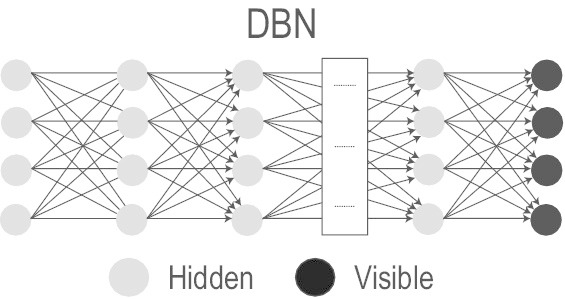	- Easy to code and works sufficiently well for just a few layers. - High performance gain by adding layers compared to multilayer perceptron. - Robust in classification.	- It can be trained greedily, one layer at a time. - Hard to deduce posterior distribution for configurations of hidden causes.
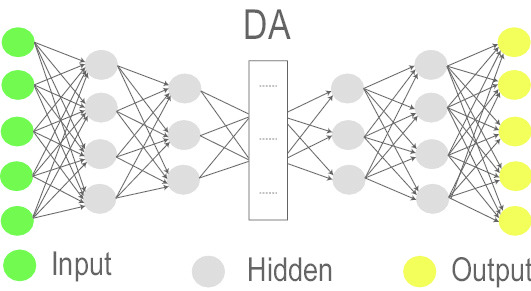	- Learn data encoding, reconstruction and generation at same time. - Training is stable without label data. - Variant: sparse, denoising and contractive DA.	- Requires pretraining stage due to the chances of vanishing error. - Each application requires redesigned and retrained the model. - The DA is sensitive to input errors.
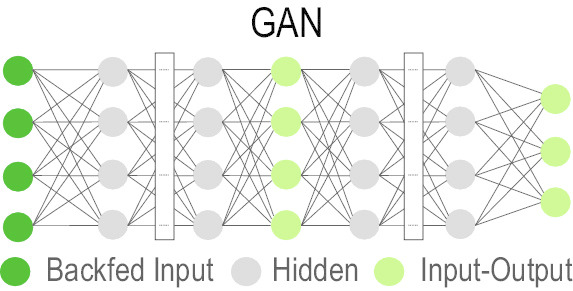	- The main benefit is data augmentation. - GAN performs unsupervised learning. - GAN learns density distributions of data.	- Difficult to train as optimizing loss function is hard and requires a lot of trial and error.
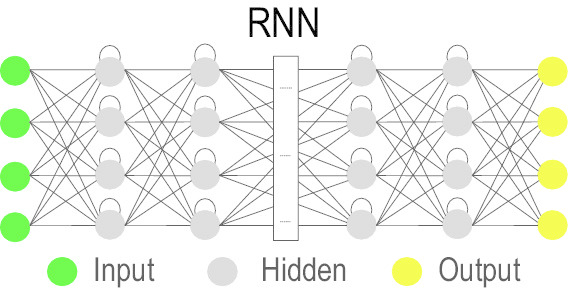	- It can process inputs of any length. - RNN can use internal memory and performs well for stream time series data.	- Computation is slow and training can be difficult. - Processing long sequences is difficult. - Prone to problems such as exploding and gradient vanishing.
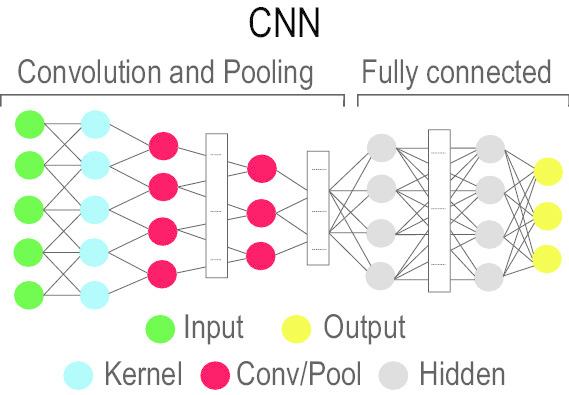	- CNN can capture hierarchical information. - CNN can share pretrained weight which is required for transfer learning. - Requires less connection compared to DNN.	- Large labelled dataset is required for training. - The working mechanism of CNN is not clear.

Legend: *DA* Deep Autoencoder, *DBN* Deep Belief Network, *RNN* Recurrent Neural Network, *DNN* Deep Neural Network, *DBM* Deep Boltzmann Machine, *CNN* Convolutional Neural Network.

## Overview of Deep Learning

In DL the data representations are learned with increasing abstraction levels, i.e., at each level more abstract representations are learned by defining them in terms of less abstract representations at lower levels [62]. Through this hierarchical learning process, a system can learn complex representations directly from the raw data [63].

Though many DL architectures have been proposed in the literature for various applications, there has been a consistent preference to use particular variants for biological data. As shown in Fig. 2, the most popular models have been identified as—Deep Neural Network (DNN), Deep Boltzmann Machine (DBM) and Deep Belief Network (DBN), Deep Autoencoder (DA), Generative Adversarial Network (GAN), Recurrent Neural Network (RNN, including LSTM), and Convolutional Neural Network (CNN). Each of these models’ architectures and their respective pros and cons are listed in Table 1. The following subsections introduce each of these most frequently used DL architectures in mining biological data.

### Deep Neural Network (DNN)

A DNN [64] is inspired by the brain’s multilevel visual processing mechanism starting with the cortical area ‘V1’ and then to area ‘V2’, and so on [65]. Mimicking this, the traditional artificial neural network or NN is extended with additional hidden layers containing nonlinear computational units in each of these hidden layers to learn a subset of the given representations. Despite its successful usage in a range of different applications, the main drawback has been the slow and cumbersome training process [66].

### [Restricted] Boltzmann Machines ([R]BM)

[R]BM represents specific probability distributions through a undirected probabilistic generative model [67]. Considered as a nonlinear feature detector, [R]BM is trained based on optimizing its parameters for a set of given observations to obtain the best possible fit of the probability distribution through a Markov chain Monte Carlo method known as Gibbs sampling [68, 69]. With symmetrical connections among subsequent units in multiple hidden layers, BM has only one visible layer. The main drawback of the standard BM is that, the learning process is computationally expensive and quite slow. Due to this, a BM requires a long period to reach equilibrium statistics [62]. However, this learning inefficiency can be solved by forming a bipartite graph (i.e. restricting to have one hidden layer and one visible layer) [67]. To extend this shallow architecture to a deep one, multiple RBMs as unitary learning elements are stacked together and this yields the following two DL architectures.

#### Deep Boltzmann Machine (DBM)

DBM [70] is a stack of undirected RBMs which supports a feedback mechanism among the layers to facilitate inference from higher-level units to propagate to lower-level units. This allows an input to be alternatively interpreted through concurrent competition at all levels of the model. Despite this powerful inference mechanism, estimating model parameters from data remains a challenge and cannot be solved using traditional gradient-based methods (e.g., persistent contrastive divergence [71]) [70]. Though this learning problem is overcome by pretraining each RBM in a layerwise greedy fashion, with outputs of the hidden variables from lower layers as input to upper layers [67], the time complexity remains high and the approach may not be suitable for large training datasets [72].

#### Deep Belief Network (DBN)

DBN [73], in contrast to the DBM, is formed by stacking several RBMs together in a way that one RBM’s latent layer is linked to the next RBM’s visible layer. As the top two layers of DBN are undirected, the connections are downward directed to its immediate lower layer [73, 74]. Thus, the DBN is a hybrid model with the first two layers as a undirected graphical model and the rest being directed generative model. The different layers are learned in a layerwise greedy fashion and fine-tuned based on required output [75]; however, the training procedure is computationally demanding.

### Deep Autoencoder (DA)

DA is a DL architecture [76] obtained by stacking a number of data-driven Autoencoders which are unsupervised elements. DA is also known as DAE and is designed to reduce data dimension by automatically projecting incoming representations to a lower-dimensional space than that of the input. In an Autoencoder, equal amounts of units are used in the input/output layers and less units in the hidden layers. (Non)linear transformations are embodied in the hidden layer units to encode the given input into smaller dimensions [77]. Despite the fact that it requires a pretraining stage and suffers from a vanishing error, this architecture is popular for its data compression capability and has many variants, e.g. Denoising Autoencoder [76], Sparse Autoencoder [78], Variational Autoencoder [79], and Contractive Autoencoder [80].

### Generative Adversarial Network (GAN)

GAN [81] is an effective generative model. Generative models perform an unsupervised learning task, where they automatically discover and learn existing patterns in data and then use that knowledge to generate new examples of the learnt pattern as if they were drawn from the original dataset. Using GAN, the problem is seen as a supervised learning problem with two strands: (i) the generator, which generates new examples as trained, and (ii) the discriminator, which classifies generated examples to two classes (real or fake). These generator and discriminator models are trained together in a zero-sum game (i.e. in an adversarial fashion) such that the examples generated by the generator model maximize the loss of the discriminator model [82, 83].

### Recurrent Neural Network (RNN)

The RNN architecture [84] is designed to detect spatio-temporal alignments in streams of data [85]. Unlike feedforward NN which performs computations unidirectionally from input to output, an RNN computes the current state’s output depending on the outputs of the previous states. Due to this ‘memory’-like property, despite learning problems related to vanishing and exploding gradients, RNN has gained popularity in many fields involving streaming data (e.g. text mining, time series, genomes, financial, etc.). In recent years, two main variants, bidirectional RNN (BRNN) [86] and Long Short-Term Memory (LSTM) [87], have also been applied [48, 88, 89].

### Convolutional Neural Network (CNN)

CNN [90] is a multilayer NN model [91] which has gained popularity in analysing image-based data. Inspired by the neurobiology of the visual cortex, the CNN consists of convolutional layer(s) containing a set of learnable filter banks and followed by fully connected layer(s). These filter banks convolve with the input data and pass the results to activation functions (e.g. ReLU, Sigmoid, and Tanh). There also exist subsampling steps in between these layers. The CNN outperforms DNNs, which as they do not scale well with multidimensional locally correlated input data. To address the scaling problem of DNNs, the CNN approach has been quite successful in analysing datasets with a high number of nodes and parameters (e.g. images). As the images are ‘stationary,’ convolution filters (CF) can easily learn data-driven kernels. Applying such CF along with a suitable pooling function reduces the features that are supplied to the fully connected network to classify. However, in case of large datasets even this can be daunting and can be solved using sparsely connected networks. Some of the popular CNN configurations include AlexNet [92], VGGNet [93] GoogLeNet [94], etc. (see Table 2 for a complete list of CNN’s variations with relevant details).

**Table 2:** Keypoints of different deep CNN architectures

Architecture	Network Design	Parameters	Key points
LeNet (1998)	LeNet-5 is the first CNN architecture with 2 convolutional and 3 fully connected layers.	0.06 million	- Feedforward NN. - Connection between layers is sparse to reduce computational complexity.
AlexNet (2012)	AlexNet has 8 layers and consists of 5 convolutional and 3 fully connected layers.	60 million	- Deeper than the LeNet and aliasing artifacts in the learned feature maps due to large filter size.
VGG-16 (2014)	VGG-16 has 13 convolutional layers (and max pooling layers) and 2 fully connected layers followed by 1 output layer with softmax activation.	138 million	- Roughly twice deeper network can be designed compared to the AlexNet. - A deeper variant of VGG-16 is VGG-19. - Computationally expensive and cannot be used with low resource systems.
Inception-v1 (2014)	Also known as GoogleNet, it has 22 layers with parameters (or 27 when pooling layers are included). Towards the end, it employs an average pooling.	5 million	- It uses sparse connections to overcome redundant information problem and omits irrelevant feature maps. - High accuracy with a reduced computational cost. - It's heterogeneous topology requires customization.
Inception-v3 (2015)	Inception-v3 has 48 layers with a number of inception modules (each consisting of pooling layers and convolutional filters with activation functions), concatenation layers and fully connected layer(s) along with dropout and softmax.	23 million	- It increases accuracy and reduces computational complexity in comparison to Inception-v1. - Reduces representational bottleneck. - Replaces large size filters with smaller ones. - It's architecture is complex and lacks homogeneity.
ResNet-50 (2015)	ResNet-50 has 50 layers with initial convolutional and max-pooling layers, and final average pooling and fully connected layers. In between, there are 3, 4, 6 and 3 residual blocks separated in 4 stages where each block contains 3 convolutional layers.	25.5 million	- It provides an accelerated training speed.↵ -Reduces the effect of Vanishing Gradient Problem. - Classifies images with high accuracy.
Xception (2016)	The Xception architecture has 36 convolutional layers forming the feature extraction base of the network. The 36 convolutional layers are structured into 14 modules, all of which have linear residual connections around them, except for the first and last modules.	22.8 million	- Xception shows small gains in classification performance on the ImageNet dataset and large gains on the JFT dataset when compared to Inception-v3.
Inception-v4 (2016)	Inception-v4 consists of two main sections: a feature extractor and a fully connected layer. The feature extractor includes various convolutional blocks such as 1 stem block, 14 inception blocks, 2 reduction blocks and a pooling layer. The inception blocks are divided in three categories, namely, A, B, and C with 4, 7, and 3 blocks, respectively.	43 million	- Deep hierarchies of features, multilevel feature representation.↵ - Learning speed is slow.
Inception-ResNet-v2 (2016)	Inception-ResNet-v2 consists of 164 layers with several convolutional blocks which include 1 stem block, 20 residual inception blocks, 2 reduction blocks and a pooling layer. The residual inception blocks are divided in three categories, namely, A, B, and C with 5, 10, and 5 blocks, respectively.	56 million	- It improves training speed.↵ - Deep hierarchies of features, multilevel feature representation.
ResNeXt-50 (2016)	ResNeXt-50 has initial convolutional and max-pooling layers, and final average pooling and fully connected layers. In between, there are 3, 4, 6 and 3 residual blocks separated in 4 stages where each block contains 3 convolutional layers. In comparison to ResNet-50, it scales up the number of parallel towers (cardinality=32) within each residual block.	25 millions	- Has homogeneous topology. ↵ - Performs grouped convolution.
DenseNet-121 (2016)	DenseNet architecture includes 4 dense blocks. Each layer in a dense block is connected to every other layer. The dense blocks, consisting of convolution, pooling, batch normalization and activation, are separated by transition layers.	8 millions	- Introduces depth or cross-layer dimension.↵ - Ensures maximum data flow between the layers in the network. ↵ - Avoids relearning of redundant feature maps.

**Table 3:** Deep learning applied to biological data

Type	Data [base/set]	DL architecture	Task
Images	ABIDE	DNN [95]	Autism disorder identification
	ADHD-200 dataset	DBN [96]	ADHD detection
	ADNI dataset	CNN [97], DBM [98], DBN [99]	AD/MCI diagnosis
	BRATS Dataset	CNN [100]	Brain pathology segmentation
	CT dataset	CNN [101]	Fast segmentation of 3D medical images
	DRIVE, STARE datasets	GAN [102]	Retinal blood vessel segmentation
	EM segmentation challenge dataset	CNN [103]	Segment neuronal membranes
		LSTM [104]	Biomedical volumetric image segmentation
	IBSR, LPBA40, and OASIS dataset	CNN [105]	Skull stripping
	LIDC-IDRI dataset	CNN [106]	Lung nodule malignancy classification
	MICCAI 2009 LV dataset	DBN [107]	Heart LV segmentation
	MITOS dataset	CNN [108]	Mitosis detection in breast cancer
	PACS dataset	CNN [106]	Medical image classification
	TBI dataset	CNN [109]	Brain lesion segmentation
Signals	BCI competition IV	DBN [110], CNN [111–113]	Motion action decoding
	DEAP dataset	DBN [114, 115]	Affective state recognition
		CNN [116]	Emotion classification
	DECAF	GAN [117]	
	Freiburg dataset	CNN [118]	Seizure prediction
	MAHNOB-HCI	DA [119]	Emotion recognition
	MIT-BIH arrhythmia database	DBN [120, 121]	ECG arrhythmia classification
	MIT-BIH, INCART, and SVDB	CNN [122]	Movement decoding
	NinaPro database	DBN [123], CNN [122]	Motion action decoding
Sequences	CullPDB, CB513, CASP datasets, CAMEO	CNN [124]	2ps prediction
	DREAM	CNN [125]	DNA/RNA sequence prediction
		DNN [126]	Predict effective drug combination
	ENCODE database	CNN [127, 128]	Gene expression identification
	ENCODE DGF dataset	CNN [129]	Predict noncoding variant of gene
	GEO database	GAN [130]	Gene expression data augmentation
	GWH and UCSC datasets	DBN [131]	Splice junctions prediction
	JASPAR database and ENCODE	CNN [132]	Predicting DNA-binding protein
	miRBoost	RNN [133]	micro-RNA Prediction
	miRNA-mRNA pairing data repository	LSTM [134]	micro-RNA target prediction
	Protein Data Bank (PDB)	DA [135]	Protein structure reconstruction
	SRBCT, prostate tumour, and MLL GE	DBN [136]	Gene/MiRNA feature selection
	sbv IMPROVER	DBN [137]	Human diseases and drug development
	TCGA database	DA [138]	Cancer detection and gene identification
		DBM [139]	
		DNN [140]	Drug combination estimation
	UCSC, CGHV Data, SPIDEX database	CNN [141]	Genetic variants identification

**Fig. 3:**
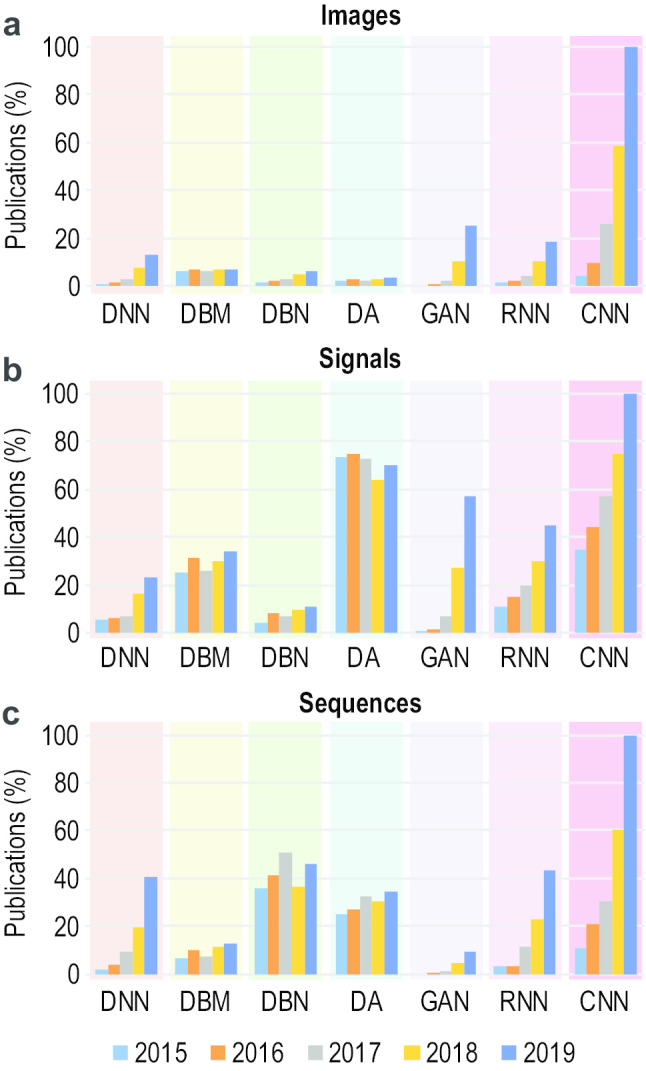
Trends in publication involving different DL architectures from 2015 to 2019 in three major types of data—images **a**, signals **b**, and sequences **c**. The numbers of papers have been normalized within each data type. However, it is noteworthy that the ratio of number of publications involving DL techniques applied to different data types (images, signals, and sequences) are approximately—1:$\frac{1}{4}$:$\frac{1}{10}$

## Deep Learning and Biological Data

Many studies have been reported in the literature which employ diverse DL architectures with related and varied parameter sets (see section 2) to analyse patterns in biological data. For most of the DL architectures, as shown in Fig. 3, the number of publications is increasing steadily over the years. A set of randomly selected representative studies from the large amount of reported literature are described below and summarized in Table 3. These studies belong to the three data types we have considered within the context of this paper, that is, images, signals, and sequences.

### Images

CNN was used by on histology images of the breast to find mitosis [108, 142] and to segment neuronal structures in Electron Microscope Images (EMI) [103]. Havaei et al. used CNN to segment brain tumour from Magnetic Resonance Imaging (MRI) [100] and Hosseini et al. used it for the diagnosis of Alzheimer’s disease (AD) from MRI [56, 97]. DBM [98] and RBM [99] were used in detecting AD and mild cognitive impairment (MCI) from MRI and Positron Emission Tomography (PET) scans. Again, CNN was used on MRI to detect neuroendocrine carcinoma [55, 74, 105]. CNN’s dual pathway version was used by Kamnitsas et al. to segment lesions related to tumours, traumatic injuries, and ischemic strokes [109]. CNN was also used by Fritscher et al. for volume segmentation [101] and by Cho et al. to find anatomical structures (Lung nodule to classify malignancy) [106] from Computed Tomography (CT) scans. DBN was applied on MRIs to detect Attention Deficit Hyperactivity Disorder [96] and on cardiac MRIs to segment the heart’s left ventricle [107]. GANs have gained popularity in image synthesis and data augmentation to reduce overfitting. GAN’s application in data augmentation and image translation has been reviewed in [143] and data augmentation in the CT segmentation tasks was done using CycleGAN [144]. GAN-based framework called MedGAN was proposed for medical image-to-image translation [145]. GAN was used as survival prediction model for chest CT scan images of patients suffering from idiopathic pulmonary fibrosis [146, 147]. GAN was also used by Halicek for synthesizing hyperspectral images from digitized histology of breast cancer cells [148].

### Signals

A stacked DA was employed to detect emotion from Electroencephalography (EEG) signals after extracting relevant features using Principal Component Analysis (PCA) and reducing non-stationary effect using covariate shift adaptation [119]. DBN was applied to decode motor imagery through classifying EEG signal [110]. For a similar purpose, CNN was used with augmented common spatial pattern features [111]. EEG signals were also classified using DA after features such as location, time, and frequency were extracted using CNN [112]. Li et al. used DBN to extract low-dimensional latent features, and select critical channels to classify affective state using EEG signals [114]. Also, Jia et al. used an active learning to train DBN and generative RBMs for the classification [115]. Tripathi et al. utilized DNN- and CNN-based model for emotion classification [116]. CNN was employed to predict seizures through synchronization patterns classification [118]. DBN [123] and CNN [122] were used to decode motion action from NinaPro database. The later approach was also used on MIT-BIH, INCART, and SVDB repositories [122]. Moreover, the Electrocardiogram (ECG) Arrhythmias were classified using DBN [120, 121] from the data supplied by MIT-BIH arrhythmia database. Zhu et al. used a GAN model with LSTM and CNN to generate ECG signals with high morphological similarity [149]. Another GAN model, RPSeqGAN, trained with SeqGAN [150] generated arrhythmic ECG data with five periods and showed high stability and data quality [151]. GAN is also used by Luo and Lu for EEG data augmentation [152]. You et al. [153] and Jiao et al. [154] utilized GAN-based model for detecting seizure using EEG signal and Driver sleepiness using EEG and Electrooculography (EOG) signals, respectively. Singh et al. proposed a new GAN framework for denoising ECG [155].

### Sequences

The stacked denoising DA has been used to extract features for cancer diagnosis and classification along with the identification of related genes from Gene Expression (GE) data [138]. GAN was also used for identifying expression patterns from GE data [156]. A template-based DA learning model was used in reconstructing the protein structures [135]. Lee et al. applied a DBN-based unsupervised method to perform autoprediction of splicing junction at Deoxyribonucleic Acid (DNA) level [131]. Combining DBN with active learning, Ibrahim et al. devised a method to select feature groups from genes or micro-Ribonucleic Acids (miRNAs) based on expression profiles [136]. For translational research, bimodal DBNs were used by Chen et al. to predict responses of human cells using model organisms [137]. Pan et al. applied a hybrid CNN-DBN model on RNAs for the prediction of RNA-binding protein (RBP) interaction sites and motifs [157], and Alipanahi et al. used CNN to predict sequence specificities of [D/R]BPs [125]. Denas and Taylor used CNN to preprocess data generated from Chromatin Immunoprecipitation followed by sequencing (ChIP-seq) and created gene transcription factor activity profiles [127]. CNN was used by Kelley et al. to predict DNA sequence accessibility [128], by Zeng et al. to predict the DBP [132], by Zhou et al. [129] and Huang et al. [141] to find non-coding gene variation, and by Wang et al. to predict secondary protein structure (2ps) [124]. Park et al. used LSTM to predict miRNA precursor [133] and Lee et al. [134] used it to predict miRNA precursors’ targets. GAN was used by Marouf et al. for the realistic generation of single-cell RNA-seq data [130], by Jiang et al. to predict disease gene from RNA-seq data [158], by Zhao et al. as a semi-supervised procedure for predicting drug target binding [159], and by Wang et al. for identifying expression patterns from GE data [156].

## Open Access Biological Data Sources

Reproducing scientific results, reported as statistically processed quantitative data or carefully selected representative qualitative data, has been facilitated greatly by data sharing initiatives [160]. In the last few decades, many open access data repositories have been made available for this purpose [161]. Indeed, many research funders and journals now require data used for studies to be made openly available for verification. To facilitate method development, here we list the leading and popular open access data repositories pertaining to the Sequences, Images, and Signals data which are summarized in Tables 4, 5, and 6, respectively.

**Table 4:** Application-wise categorization of open access data repositories and datasets pertaining to [bio/medical/health/clinical] images

Application	Name	Description	Ref.
Bio/medical image processing and analysis	CCDB	High-resolution 2/3/4-D light and electron microscope images	[162]
	CIL	Cell image datasets and cell library app.	[163]
	Euro Bioimaging	Biological and biomedical imaging data	[164]
	HAPS	Microscopic image of human cells and tissues	[165]
	IDR	Viewing, analysis, and sharing of multi-D image data	[166]
	SMIR	Post-mortem CT scans of the whole body	[167]
	TCIA	CT, MRI, and PET images of cancer patients	[168]
	TMA	Microscopic tissue images of human	[169]
	UCSB BioSeg	2D/3D cellular, subcellular and tissue images	[170]
Disease detection and diagnosis	ABIDE	Autism brain imaging datasets	[171]
	ADHD-200	fMRI/anatomical datasets fused over the 8 imaging sites	[172]
	ADNI	MCI, early AD and elderly control subjects’ diagnosis data	[173]
	BCDR	Multimodal mammography and ultrasound scan data	[174]
	Kaggle CXRayP	Chest X-ray scans for pneumonia	[175]
	MITOS	Breast cancer histological images	[176]
	NAMIC	Lupus, brain, prostate MRI scans	[177]
	nCOV-CXray	COVID-19 cases with chest X-ray/CT images	[178]
	Neurosynth	fMRI datasets and synthesis platform	[179]
	NIH	Labelled chest X-ray images with diagnoses	[180]
	OASIS	MRI datasets and XNAT data management platform	[181]
	Open NI	Imaging modalities and brain diseases data	[182]
	SMIR	CT of human temporal bones	[183]
Neuroimage processing and analysis	IXI	It provides neuroimaging data and toolkit software	[184]
	LPBA40	Maps of brain regions and a set of whole-head MRI	[185]
	NeuroVault.org	API for collecting and sharing statistical maps of brain	[186]
	NITRC	MRI, PET, SPECT, CT, MEG/EEG and optical imaging	[187]
	OpenfMRI	Multimodal MRI and EEG datasets	[188]
	UK data service	fMRI dataset	[189]
Segmentation	DRIVE	Digital Retinal Images diabetic patient	[190]
	IBSR	Segmentation results of MRI data	[191]
	STARE	The dataset contains raw/labelled retinal images	[192]

Legend: *CXRayP *Chest X-ray Pneumonia, *JHDTI *Johns Hopkins Diffusion Tensor Imaging

### Images

Table 4 lists the leading open access data sources including databases and individual datasets that provide access to data pertaining to biological image research. For the sake of simplicity, these sources have been grouped to four broad application areas—[bio/medical] image processing and analysis, disease detection and diagnosis, neuroimage processing and analysis, and segmentation—and these are briefly described below.

#### Bio/Medical Image Processing and Analysis

The Cell Centered Database (CCDB) [162] collection provides high-resolution 3-D light and electron microscopic reconstructions of cells and subcellular structures. It also contains [2/3/4]-D protein distribution and structural information from a number of different microscopic image acquisition systems.

Another image library, called the Cell Image Library (CIL) [163], presents more than 10,000 unique datasets and 20 TB of images, videos, and animations data. These data belong to a wide diversity of organisms, cell types, and cellular processes.

The Euro Bioimaging [164] database provides biological and biomedical imaging data aiming to provide collaboration among different stakeholders including scientists, industry, national and European authorities. Its mission is to give access and services to state-of-the-art imaging techniques and bioimaging data for scientists in Europe and beyond. Euro Bioimaging also includes image analysis tools.

The HAPS is a histology image database [165] contains medium-/high-resolution photograph of microscopic image of human cells and tissues which are free of any copyright. Another image database, the Image Data Resource (IDR) [166], contains individual datasets of cellular and tissue images. Various categories of images include time-lapse imaging, protein localization studies, digital pathology imaging, yeast study, human high-content screening, etc. It is also public API which facilitates viewing, analysis, and sharing of multi-D image data for cell biology.

The SICAS Medical Image Repository (SMIR) is an image repository for medical research purpose. Two of their featured collections include post-mortem full-body CT [167] scan of 50 anonymized subjects of different age groups and gender, and CT, micro-CT, segmentation, and shape models of the cochlea [183].

The Cancer Imaging Archive (TCIA) [168] contains CT, MRI, and nuclear medicine (e.g. PET) images for clinical diagnostic, biomarker, and cross-disciplinary investigation. The Stanford Tissue Microarray Database (TMA) [169] is a source for annotated microscopic tissue images and associated expression data. The data can be used for studying cell biology. The UCSB bio-segmentation benchmark dataset [170] contains 2/3-D cellular, subcellular, and tissue images. These datasets can be used for segmentation and classification task.

#### Disease Detection and Diagnosis

A large amount of imaging data has been acquired from patients with neurological disorders. The Autism Brain Imaging Data Exchange (ABIDE) [171] database includes autism brain imaging datasets for studying the autism spectrum disorder. The other dataset pertains to the Attention Deficit Hyperactivity Disorder (ADHD) [172] and includes 776 resting-state fMRI and anatomical datasets which are fused over the 8 independent imaging sites. The phenotypic information includes age, sex, diagnostic status, measured ADHD symptom, intelligence quotient, and medication status. Imaging-based diagnostic classification is the main aim of the ADHD 200 dataset. The ADNI (Alzheimer’s Disease Neuroimaging Initiative [173]) is a popular database and contains neuroimaging datasets from neurodegenerative diseases, in particular, AD, MCI, early and late AD and elderly control subjects. The datasets offered by this repository are mainly dedicated for development of novel methods for diseases related to AD. Another dataset focusing on AD is the Open Access Series of Imaging Studies (OASIS) [181] dataset. This contains MRI datasets and open-source data management platform (XNAT) to study and analyse AD. Neurosynth [179] is yet another database which includes fMRI literature (with some datasets) and synthesis platform to study brain structure, functions, and disease. On the other hand, the Open Neuroimaging (Open NI) [182] dataset contains imaging modalities and brain diseases data which can be used to study decision support system for disease identification.

The recent novel coronavirus disease or COVID-19 pandemic has attracted a number of researchers to focus their attention on the detection of the novel coronavirus disease. The NIH [180]

nCOV chest X-ray database [178] contains COVID-19 cases with chest X-ray/CT images. The data can be used for identifying bacterial vs viral vs COVID-19 pneumonia. Similar chest X-ray datasets [175] are hosted by Kaggle which include chest X-ray scans data for detecting traditional viral and bacterial pneumonia.

Breast cancer is also another important disease which can be addressed through imaging and this has attracted a number of databased hosting breast cancer images.

The Breast Cancer Digital Repository (BCDR) [174] database contains multimodal mammography and ultrasound scan and patient history data collected from 1734 anonymized patients. The data can be used for disease detection and diagnosis methods. Another dataset, MITOS [176], contains breast cancer histological images (haematoxylin and eosin stained slides). The detection of mitosis and evaluation of nuclear atypia are key uses.

#### Neuroimage Processing and Analysis

The Information eXtraction from Images (IXI) dataset [184] provides 600 MRI images from healthy subjects to study brain functions. These images saved in NIFTI file format and were acquired using protocol—T1, T2, proton-density weighted images; magnetic resonance angiography images; and diffusion weighted images. These images have been collected from three different hospitals in London, UK. Another database, called the Loni Probabilistic Brain Atlas (LPBA40) [185], contains maps of brain anatomic regions of 40 human volunteers. Each map generates a set of whole-head MRI, whereas each MRI describes to identify 56 structures of brain, most of them lies in the cortex. The study of skull-stripped MRI volumes, and classification of the native-space MRI, probabilistic maps are key uses of LPBA40. The NeuroVault.org [186] is a web-based repository (API) for collecting and sharing statistical maps of the human brain to study human brain regions. The Neuroimaging Informatics Tools and Resources Clearing house (NITRC) [187] provides range of imaging data from MRI to PET, SPECT, CT, MEG/EEG, and optical imaging for analysing functional and structural neuroimages. The Open fMRI [188] dataset contains MRI images acquired using different modalities including diffusion-weighted, T1-weighted magnetization prepared rapid acquisition with gradient echo (MPRAGE) MRI, and multiecho fast low-angle shot (FLASH) MRI. It also contains biosignal datasets to study brain regions and its functions. These can be used as a benchmark dataset in order to differentiate outcome from various neuroimaging analysis tools. The UK data service [189] contains T1/2, diffusion tensor imaging, and fMRI datasets from 22 patients suffering from brain tumours which can be useful for studying brain tumour surgical planning.

#### Segmentation

Segmentation is an important step in any image processing pipeline. Many datasets mentioned above can be used for segmentation purposes.

Focusing on eye diseases, the Digital Retinal Images for Vessel Extraction (DRIVE) contains JPEG Compressed retinal images of 400 diabetic patients between 25-90 years old. This dataset can be used to understand segmentation of blood vessels in retinal images and identify diabetic retinopathy. Another dataset called STructured Analysis of the Retina (STARE) was initiated in 1975. The project contains datasets of 400 raw retinal images, 10 labelled images of artery/vein, and 80 images with ground truth. Each image is annotated and features are shown in image by the expert. The dataset can be used for blood vessel segmentation and optic nerve detection.

The Internet Brain Segmentation Repository (IBSR) gives segmentation results of MRI data. Development of segmentation methods is the main application of this IBSR.

**Table 5:** Application-wise categorization of open access data repositories and datasets pertaining to biological signals

Application	Name	Description	Ref.
Anomaly detection	SAD mc-EEG	Multichannel EEG data for sustained-attention driving task	[193]
	TUH EEG Corpus	Repository for EEG datasets, tools and documents	[194]
	MIT-BIH-ARH	ECG database containing 48 recordings	[195]
	PTB D-ECG	ECG database containing 549 recordings	[196]
	TELE ECG	250 ECG recordings with annotated QRS and artifact masks	[197]
Human–Machine Interfacing	BNCI	Various BMI signal datasets	[198]
	EMG DataRep	Various EMG datasets	[199]
	Facial sEMG	Contains EMG data from 15 participants	[200]
	NinaPro database	Kinematic as well as the sEMG data of 27 subjects	[201]
Emotion/affective state detection	DEAP	Simultaneously recorded EMG/EEG data	[202]
	DECAF	MEG, hEOG, ECG, trapezius muscle EMG, face video data	[203]
	Imagine	EEG datasets of 31 subjects while listening voice	[204]
	MAHNOB-HCI	EMG, ECG, and respiration and skin temperature data	[205]
	SEED	EEG dataset for emotion and vigilance	[206]
Motor imagery classification	EEG-BCI-MI	EEG signals from 13 subjects with 60,000 MI examples	[207]
	EEG-MI-BCI	EEG data from BCI for MI tasks	[208]
	EEG-MMI	EEG data from PhysioNet for MI task	[209]
Neurological condition evaluation	V-P300 BCI	16-electrode dry EEG from 71 subjects (SP mode)	[210]
		32-electrode wet EEG from 50 subjects (SP mode)	[211]
		32-electrode wet EEG from 38 subjects (MPC mode)	[212]
		32-electrode wet EEG from 44 subjects (MPCC mode)	[213]
Signal processing and classification	BCI competition	EEG, ECoG, and MEG data from a range of BCI applications	[214]
	BCI-NER challenge	56 channel EEG dataset decoded by a P300 speller	[215]
	DRYAD	EEG datasets of 13 subjects recorded under various conditions	[216]
	PhysioNet	Various EEG, ECG, EMG and sEMG datasets	[217]
	UCI ML	Various ECG, EMG, sEMG datasets	[218]

Legend:* MI* Motor Imagery, *MMI* Motor Movement/Imagery, *ERP* Event-Related Potentials, *SADmc-EEG* Sustained-Attention Driving multichannel EEG, *V-P300* Visual P300, *SP* Single Player, *MP* Multiplayer, *BCI-SSVEP* Steady State Visual Evoked Potentials, *EMG DataRep* EMG Dataset Repository, *ARH* Arrhythmia, *D-ECG* Diagnostic ECG

### Signals

Table 5 lists leading open access data repositories and datasets (also referred as data sources) pertaining to biological signals. These sources are broadly mapped to six application areas—anomaly detection, human–machine interfacing which includes brain–machine interfacing as well as rehabilitation research, emotion/affective state detection, motor imagery classification, neurological condition evaluation, and signal processing and classification—which are described in the following subsections.

#### Anomaly Detection

Anomaly detection is one of the major application areas in which scientists have devoted much efforts. In this process, a number of open access data sources, largely containing EEG and ECG data, have been frequently used.

Starting with the EEG signals, the SAD mc-EEG [193] dataset contains 32 channel EEG signals from 27 subjects recorded while they were test-driving. That is, signals were acquired when each subject attended two 90-minute virtual reality session for sustained-attention driving.

The TUH EEG corpus [194] is also an open-source clinical EEG data repository for clinical EEG data, tool and documentation. The major datasets include seizure detection, abnormal EEG, EEG with artifacts (introduced by eye movement, chewing, shivering, electrode pop, electrode static, and lead artifacts, and muscle artifacts), EEG for epilepsy, etc.

Regarding the ECG signals, the MIT-BIH arrhythmia [195] arrhythmia database includes 2-channel ambulatory ECG recording taken from 47 subjects for studying arrhythmia. There are 48 complete ECG recordings and about 24 recordings are freely available. The PTB diagnostic ECG database [196] comprises 549 ECG recordings taken from 290 subjects of age ranged from 17 to 87 years using conventional 12 leads and 3 Frank lead ECG recorder. Each recording includes 15 signals coming from these leads and each subject was represented in 1 to 5 records. Both the datasets can be used for anomaly detection. Another ECG dataset, the TELE-ECG dataset [197] includes 250 ECG records with annotated QRS and artifact masks. It also includes QRS and artifact detection algorithms to study QRS and detect artifacts from ECG signals.

#### Human–Machine Interfacing

The application area of Human–Machine Interfacing focuses on [body and brain]–machine interfacing and rehabilitation. This is done largely through Electromyography (EMG) and sometimes with EEG signals.

The BNCI Horizon 2020 database contains more than 25 datasets such as stimulated EEG datasets, Electrocorticography (ECoG)-based BCI datasets, Event Related Potential (ERP)-based BCI datasets, mental arithmetic, motor imagery (extracted from EEG, EOG, fNIRS, EMG) datasets, EEG/EOG datasets of neuroprosthetic control, speller datasets. Modelling and designing of BMI devices are the key application of this database. While the BNCI contains a variety of signals, the EMG Datasets Repository [199] includes single/multifinger movements datasets of 2 channels, 10 classes and 8 channels, 15 classes; single-/multifinger pressure on a steering wheel; EMG controlled multifunctional upper-limb prostheses and EMG pattern recognition datasets.

For surface EMG (sEMG), the facial sEMG dataset contains facial sEMG signals from the muscles corrugator supercilii, zygomaticus major, orbicularis oris, orbicularis oculi, and masseter. Archived data are from 15 participants (8 females and 7 males) aged between 26 and 57 years (mean age $40.7 \pm 9.6$ years). These data can be used for rehabilitation research. Also, the NinaPro database includes kinematic as well as sEMG data of 27 subjects, while these subjects were moving finger, hand, and wrist. These data can be employed to study biorobotics and activity detection.

#### Emotion/Affective State Detection

Emotion and affective state detection has been a very active research field over the years. A combination of different signals has been utilized in detecting emotion and affective states, and a number of data sources providing these signals are described below.

A Database for Emotion Analysis using Physiological Signals (DEAP) provides various datasets for analysing the human affective states. It provides EEG and sEMG signals of 32 volunteers, while they were watching music videos to analyse the affective states. These volunteers also rated the video, and the front face was also recorded for 22 volunteers. DECAF is a multimodal dataset for decoding user physiological responses to affective multimedia content. It contains magnetoencephalogram (MEG), horizontal electrooculogram (hEOG), ECG, trapezius muscle EMG, and near-infrared face video data to study physiological and mental states. Another multimodal dataset is the MAHNOB-HCI [205] dataset which includes ECG, respiration, and skin temperature data in addition to 32-channel EEG signals from 30 subjects, while they were watching movie clips and photos. The different sensors were synchronized to record a synchronized multimodal dataset. The subjects were asked to label their own emotion state.

On the other hand, the Imagined Emotion [204] dataset provides EEG signals recorded when subjects were listening to voice recording. The SJTU Emotion EEG Dataset [206] contains three individual datasets (SEED, SEED-IV and SEED-VIG) of EEG signals. In the SEED dataset EEG signals were recorded, while the subjects were watching movie clips and annotated their emotional state as positive, negative and neural. In case of SEED-IV, four emotional states such as happy, sad, fear, and neutral were annotated, whereas the SEED-VIG dataset contains EEG signals related to vigilance when the subjects were driving.

#### Motor Imagery Classification

Motor imagery (MI) is yet another very active area of research. As an outcome of a large number of community contributors, many datasets have been developed from which the popular ones are described below.

The electroencephalographic brain–computer interface mental imagery (EEG-BCI-MI) [207] dataset contains 60 hours of EEG recording from 13 subjects and 75 experiments. This contains around 60,000 mental imagery examples which is approximately 4.8 hours of EEG recordings (with 4600 MI examples) per participant. The datasets can be used for the rehabilitation of patients having movement disorders. Another EEG dataset for MI brain–computer interface (EEG-MI-BCI) [208] contains EEG signals with 3-D electrode location and EEG for non-task-related states as well. The dataset was recorded from 52 participants which also contains [physio/psyco]logical data and EMG signals in addition to the EEG. The dataset can be employed to find the human factors which influence MI BCI performances. Yet another EEG signal centric dataset is called EEG motor movement/imagery (EEG-MMI) dataset [209] and incorporates 1500 (1–2 minutes) EEG recordings taken from 109 volunteers. The dataset can be used in designing BCI systems for rehabilitation purposes.

#### Neurological Condition Evaluation

A number of visual P300-based datasets are available with open-access attributes to perform a range of neurological condition evaluation. These datasets, V-P300 BCI, are composed of data recorded using dry or wet electrode with 16 or 32 channels while the subjects were playing the Brain Invaders game [219]. These datasets were recorded using different playing modalities such as single player (16 dry electrodes [210] from 71 subjects and 32 wet electrodes [211] from 50 subjects), multiplayer in collaborative mode (32 wet electrodes from 38 subjects [212]), and multiplayer cooperation and competition mode (32 wet electrodes from 44 subjects [213]).

#### Signal Processing and Classification

To solve various signal processing and classification problems, a number of datasets have been made available under open-access. Most of these problems are released to the community in the form of challenges with relevant datasets to solve them. The competitions during the BCI meetings have served this purpose for several years and have released datasets (the BCI competition datasets [214]) which are still available with relevant problem statements and sample codes for others to use. The challenge dataset provided by the IEEE Neural Engineering Conference (NER2015) is known as BCI-NER dataset [215]. This dataset was mainly intended for methodological development of an error detection algorithm suitable for the P300-based BCI systems. The BCI competition datasets include EEG datasets (e.g., cortical negativity or positivity, feedback test trials, self-paced key typing, P300 speller paradigm, motor/mental imagery data, continuous EEG, EEG with eye movement), ECoG datasets (e.g., finger movement, motor/mental imagery signals in the form of EEG/ECoG), and MEG dataset (e.g., wrist movement). These datasets can be used for signal processing and classification methods for BMI. Similarly, the BCI-NER Challenge [215] dataset provides 56-channel EEG signals from 26 subjects using a P300 speller.

In addition to the datasets released for challenges and competitions, there are repositories which provide rich datasets for this application area. The DRYAD [216] is a versatile repository which has been recently unveiled. It contains a range of EEG recorded datasets when 19 subjects listen to natural speech time-reversed speech, cocktail party attention, and noisy audiovisual speech. The PhysioNet repository [217] contains a large number of neuroelectric and myoelectric datasets. As the name suggests, it is mainly for physiological data. These datasets mainly pertain to signals such as EEG, ECoG, EMG, and ECG and are acquired from many diverse experimental settings. The UCI ML repository [218] contains a large number of diverse datasets with direct application to machine learning methods. Some relevant biosignal datasets include ECG, EEG, and (s)EMG signals from diverse experimental and physiological conditions.

**Table 6:** Application-wise categorization of open access data repositories and datasets pertaining to Omics data

Application	Name	Description	Ref.
Bioassay analysis and drug design	COVID-19	Gene sequence, pathway, and bioassay datasets of COVID-19	[220]
	PubChem	Contains compound structures, molecular datasets, and tool	[221]
Genetic disorder analysis	Cancer GeEx	Different cancer genome datasets	[222]
	IGDD	Mutation data on common genetic diseases	[223]
	TCGA	Contains cancer genome data	[224]
	BDTNP	3D Gene expression, DNA-binding data and ChAcD	[225]
Nucleic acid research	ENCODE	Human genome dataset	[226]
	ESP	Contains sequencing data	[227]
	GEO	Contains high-throughput gene expression and functional genomics datasets	[228]
	gnomAD	Large-scale exomes and genomes sequencing data	[229]
	GTEx	Gene expression datasets	[230]
	Harmonizome	Collection of genes and proteins datasets	[231]
	INSDC	Contains nucleotide sequence data	[232]
	IGSR	Genome data of various ethnicities, age and sex	[233]
	JASPAR	Transcription factor DNA-binding preferences dataset	[234]
	NIHREM	Human genome datasets	[235]
	NSD	Includes omics and health science data	[236]
	SysGenSim	Bioinformatics tools and gene sequence dataset	[237]
Protein structure analysis	PDB	Proteins, nucleic acids, and complex assemblies data	[238]
	SCOP2	Contains structural classification of proteins	[239]
	SCOPe		[240]
	UCI MB	2ps and splice–junction gene sequences	[241]
Signal transduction pathway study	NCI Nature	Molecular interactions and reactions of cells	[242]
	NetPath	Signal transduction pathways in humans	[243]
	Reactome	Database for reactions, pathways and biological processes	[244]
Single-cell omics	miRBoost	The genomes of eukaryotes containing at least 100 miRNAs	[245]
	SGD	Provides biological data for budding yeast and analysis tool	[246]

### Sequences

Table 6 lists the leading popular open access data sources pertaining to the various omics-related researches which include genomics, proteomics, and metabolomics. Grouped to six broad application areas, namely, bioassay analysis and drug design, genetic disorder analysis, nucleic acid research, protein structure analysis, signal transduction pathway study, and single-cell omics, the following subsections provide brief discussions about the leading open access omics data sources.

#### Bioassay Analysis and Drug Design

Since December 2019, the world has experienced a pandemic caused by the SARS-CoV-2 (COVID-19) virus. Triggered by the necessity to facilitate the ongoing researches, the SARS-CoV-2 [220] dataset provides gene sequence, proteins, pathway, and bioassay for SARS-CoV-2 along with compounds used in clinical trials. This dataset can be used for studying biological/chemical process and drug design.

The PubChem database [221] contains millions of compound structures and descriptive datasets of chemical molecules and their activities against biological assays. Maintained by the National Center for Biotechnology Information of the United States National Institutes of Health, it can be freely accessed through a web user interface and downloaded via FTP. It also contains software services (such as plotting and clustering). It can be used for [gen/prote]-omics study and drug design.

#### Genetic Disorder Analysis

The cancer gene expression (GE) [222] serves as a small repository containing several cancer GE datasets which can be employed for designing tool/algorithm for cancer detection. The cancer genome atlas (TCGA) [224] repository contains more than 2.5 petabytes of genomic, epigenomic, transcriptomic, and proteomic data. It contains data about 33 different cancer types and over 20,000 samples. These data are generated by the National Cancer Institute and the National Human Genome Research Institute. This repository is used in facilitating genomic study for improving the prevention, diagnosis, and treatment of cancer. To analyse region-specific diseases, the Indian Genetic Disease Database (IGDD) [223] tracks mutations in the normal genes for genetic diseases reported in India.

#### Nucleic Acid Research

The Berkeley Drosophila Transcription Network Project (BDTNP) [225] database contains datasets pertaining to 3D Gene expression data, in vivo and in vitro DNA-binding data as well as Chromatin Accessibility data (ChAcD). Research on GE and anomaly detection is the key application of the datasets provided by this database.

The Encyclopedia of DNA Elements (ENCODE) [226] is a whole-genome database curated by the ENCODE Consortium. It contains a large number of datasets pertaining to functional genomics and characterization data including meta-data of human, worm, mouse, and fly. Another database, called the Exome Sequencing Project (ESP) [227], includes genome datasets which can be used to find lung and blood disorders and their management and treatment. The Gene Expression Omnibus (GEO) [228] is an open-access functional genomics (microarray and sequence) data repository. This database can be used for functional genomic and epigenomic studies such as genome methylation, chromatin structure, and genome–protein interactions. It is supported by the National Center for Biotechnology Information at the National Library of Medicine of the USA [228]. The Genome Aggregation Database (gnomAD) [229] database contains large-scale exome and genome sequencing data from different sequencing projects. The dataset can be used for disease diagnosis and genetic studies. The Genotype-Tissue Expression (GTEx) [230] database contains GE datasets of 54 healthy tissue sites collected from 1000 subjects and histology images. It also includes samples from GTEx biobank.

The Harmonizome [231] database provides details about genes and proteins from 114 datasets provided by 66 online resources with 71927784 associations between 295496 attributes and 56720 genes. The International Nucleotide Sequence Database [232], popularly known as INSDC, corroborates biological data from three major sources: i) DNA Databank of Japan [247], ii) European Nucleotide Archive [248], and iii) GenBank [249]. These sources provide the spectrum of data raw reads, though alignments, and assemblies to functional annotation, enriched with contextual information relating to samples and experimental configurations. Similar to this, the International Genome Sample Resource (IGSR) [233] includes genome sequencing data from 1000 genomes project. The genome data was taken from people of various ethnicities, age, and sex with the final dataset contains gene sequencing data from 2,504 individuals from 26 populations. These data can be used for disease diagnosis and genetic studies. Also, the SysGenSim [237] database includes bioinformatics tool, and Pula-Magdeburg single-gene knockout, StatSeq, and DREAM 5 benchmark datasets for studying Gene Sequence.

JASPAR [234] is a database for transcription factor DNA-binding profile. The data spans through six different taxonomic groups covering Vertebrata, Nematoda, Insecta, Plantae, Fungi, and Urochordata. The database can be used for translational genomics research.

The NIH Roadmap Epigenomics Mapping repository (NIHREM) [235] includes 2,804 datasets, i.e., 1,821 histone modification, 360 DNase, 277 DNA methylation, and 166 RNA-Seq datasets. The repository provides 3,174-fold 150.21 billion mapped sequencing the human and tools for analysing these datasets. It can be used for stem cell mapping and selection of tissues that are responsible for human disease. Also, the database known as Nature scientific data (NSD) [236] includes datasets pertaining to omics, taxonomy and species diversity, mathematical and modelling resources, cytometry, organism-focused resources, and health science data. This can be used for studying and modelling different aspects of genomics.

#### Protein Structure Analysis

The Protein Data Bank (PDB) [238] contains 3D structural data proteins and nucleic acids. These data are obtained tools such as X-ray crystallography, NMR spectroscopy, and cryo-electron microscopy. It includes more than 135 thousand data of proteins, nucleic acids, and complex assemblies. These can be used to understand all aspects of biomedicine and agriculture.

Structural classification of proteins (SCOP) is a repository which hosts manually classified protein structure datasets. The classification was done based on amino acid sequences and their structural similarity. The main objective is to find the evolutionary relationship between the proteins. Currently two versions of SCOP are maintained. The SCOP Version 2 (SCOP2) [239] is the up-to-date SCOP database released at the first quarter of 2020. In contrast, the SCOP-extended (SCOPe) [240] is an extended version of the original SCOP maintained by UC Berkeley. SCOPe includes many new classified protein structures via a fusion of manual and automation curation.

Molecular Biology Databases at the UCI (UCI MB) contain three individual databases: i) Secondary Protein Structure [241], which is a bench repository that classifies secondary structure of certain globular proteins; ii) Splice–Junction Gene Sequences [250], which contain primate splice–junction gene sequences (DNA) with associated imperfect domain theory; and iii) Promoter Gene Sequences [251], which contain E. coli promoter gene sequences (DNA) with partial domain theory. Objectives include i) sequencing and predicting the secondary structure of certain proteins; ii) studying primate splice–junction gene sequences (DNA) with associated imperfect domain theory; iii) studying E. Coli promoter gene sequences (DNA) with partial domain theory.

#### Signal Transduction Pathway Study

The NCI–Nature Pathway Interaction Database [242] hosts cellular signalling (molecular interactions/reactions) pathways in humans. The database can be employed for cancer research. The database was created by the U.S. National Cancer Institute, NIH, with the collaboration of Nature Publishing Group and published in the last quarter of 2006. Another database, NetPath [243], also contains signal transduction pathways in humans. Created jointly by Johns Hopkins University and the Institute of Bioinformatics (IOB) in India; it includes 45 signalling pathway ranging from protein–protein interactions to enzyme–protein substrate reactions including 10 major pathway of immune system and 10 pathway relevant to cancer regulation. The other one, Reactome [244], is an open access database hosting biological pathways of metabolic processes to hormonal signalling in humans. Created through a collaboration between North America and Europe, it can be used for cancer research and treatment.

#### Single-cell Omics

The miRBoost dataset [245] contains the genomes of eukaryotes containing at least 100 miRNAs. This dataset is used for studying post-transcriptional gene regulation (PTGeR) and miRNA-related pathology. Saccharomyces Genome Database (SGD) [246] also provides complete biological information for the budding yeast *Saccharomyces cerevisiae*. They also give an open-source tool for searching and analysing these data and thereby enable the discovery of functional relationships between sequence and gene products in fungi and higher organisms. The study of genome expression, transcriptome, and computational biology is the main function of the SGD.

## Open-Source Deep Learning Tools

Due to surging interest and concurrent multidisciplinary efforts towards DL in the recent years, several open-source libraries, frameworks, and platforms have been made available to the community. However, for a new user of these tools to mine biological data, it is not always straightforward to know their characteristics, advantages, and disadvantages. In this process, one of the main hurdles for a new analyst is to select the appropriate DL architecture/model and relevant library providing suitable implementations of the selected architecture. Towards introducing a beginner to the field of biological data analysis using these open-source tools, this section describes the tools in a tutorial style indicating their characteristics, pros, and cons. The focus of the section has been to review and summarize the most popular open-source tools, which aim to facilitate the technological developments for the community. This comprehensive collection contains tools (also developed by individuals) which are well maintained with a reasonable amount of implemented algorithms (i.e., deep learning architectures). For the sake of brevity, the individual publication references of the tools are omitted and interested readers may consult them at their respective websites from the provided URLs.

Table 7 summarizes the main features and differences of the various tools. To measure the impact and acceptability of a tool in the community, we provide GitHub-based measures such as numbers of Stars, Forks, and Contributors. These numbers are indicative of the popularity, maturity, and diffusion of a tool in the community.

**Table 7:** Summary of Open-Source Deep Learning Tools (* as of July 2020)

Tool	Platform	Language(s)	Stars*	Forks*	Contrib.*	Supported DL Architecture
Caffe^b^	L, M, W, A	Py, C++, Ma	30100	18200	266	CNN, RNN, GAN
Chainer^c^	L	Py	5300	1400	251	DA, CNN, RNN, GAN
DL4j^a^	L, M, W	Ja	11500	4800	32	DA, CNN, RNN, RBM, LSTM, GAN
DyNet^a^	L	C++	3000	687	117	CNN, RNN, LSTM
H_2_O^a^	L, M, W	Ja, Py, R	4700	1700	132	CNN, RNN
Keras^c^	L, M, W	Py	47500	18000	816	CNN, RNN, DBN, GAN
Lasagne^a^	L, M	Py	3700	980	68	CNN, RNN, LSTM, GAN
MCT^c^	W	C++	16720	4400	197	CNN, DBN, RNN, LSTM
MXNet^a^	L, M, W, A, I	C++	18500	6600	780	DA, CNN, RNN, LSTM, GAN
Neon^a^	L, M	Py	3800	846	78	DA, CNN, RNN, LSTM, GAN
PyTorch^b^	L, M	Py	37400	9500	1345	CNN, RNN, LSTM, GAN
Singha^a^	L, M, W	Py, C++, Ja	2000	499	46	CNN, RNN, RBM, DBM
TensorFlow^a^	L, M, W	Py, C++	14300	80600	2450	CNN, RNN, RBM, LSTM, GAN
TF.Learn^c^	L, M	Py, C++	9400	2400	120	CNN, BRNN, RNN, LSTM, GAN
Theano^b^	L, M, W	Py	9103	2500	332	CNN, RNN, RBM, LSTM, GAN
Torch^b^	L, M, W, A, I	Lu, C, C++	8495	2400	130	CNN, RNN, RBM, LSTM, GAN
Veles^a^	L, M, W, A	Py	891	185	10	DA, CNN, RNN, LSTM, RBM

*L* Linux/Unix, *M* MacOSX, *W* Windows, *A* Android, *I* iOS, *CP* Cross-platform, *Py* Python,* Ja* Java, *Lu* Lua, *Ma* Matlab

*GitHub parameters (as of 1 April. 2020)

^a^Apache2 License

^b^BSD License

^c^MIT License

### Caffe

Caffe (http://caffe.berkeleyvision.org/) is scalable, written in C++ and provides bindings for Python as well as MATLAB. Dedicated for experiment, training, and deploying general purpose DL models, this framework allows switching between development and deployment platforms. Targeting computer vision applications, it is considered as the fastest implementation of the CNN.

**Pros.**

- Easy to deploy;
- Pretrained models are available;
- Faster training speed;
- Used for feedforward networks.

**Cons.**

- Requires writing code for generating new layers;
- Less support for recurrent networks;
- No support for distributed training.

### Chainer

Chainer (http://chainer.org/) is a DL framework provided as Python library. Besides the availability of popular optimization techniques and NN related computations (e.g., convolution, loss, and activation functions), dynamic creation of graphs makes Chainer powerful. It supports a wide range of DL architectures including CNN, GAN, RNN, and DA.

**Pros.**

- One of the tools for leading dynamic computation graphs/networks;
- Notably faster than other Python-oriented frameworks.

**Cons.**

- Open Computing Language framework/Open Multi-Processing API is not supported.

### DeepLearning4j

DeepLearning4j (DL4J, https://deeplearning4j.org/), written in Java with core libraries in C/C++, is a distributed framework for quick prototyping that targets mainly non-researchers. Compatible with JVM supported languages (e.g., Scala/Clojure), it works on distributed processing frameworks (e.g., Hadoop and Spark). Through Keras (see section 5.6) as a Python API, it allows importing existing DL models from other frameworks. It allows creation of NN architectures by combining available shallow NN architectures.

**Pros.**

- Supports integration with Big Data frameworks Apache Spark and Hadoop;
- Supports distributed GPU and CPU platforms and capable to work with tensor.

**Cons.**

- Open Computing Language framework is not supported;
- GUI is supported for workflow and visualization.

### DyNet

The DyNet library (https://dynet.readthedocs.io/), written in C++ with Python bindings, is the successor of the ‘C++ neural network library’. In DyNet, computational graphs are dynamically created for each training example; thus, it is computationally efficient and flexible. Targeting NLP applications, its specialty is in CNN, RNN, and LSTM.

**Pros.**

- Designed to be efficient for running on CPU or GPU.
- Dynamic computation graph like PyTorch and Chainer.

**Cons.**

- In terms of TensorFlow, limited functions are available.

### H$_2$O

H$_2$O (http://www.h2o.ai) is an ML software that includes DL and data analysis. It provides a unified interface to other DL frameworks like TensorFlow, MXNet, and Caffe. It also supports training of DL models (CNN and RNN) designed in R, Python, Java, and Scala.

**Pros.**

- Due to its in-memory distributed parallel processing capacities, it can be used for real-time data;
- GUI is supported (called Flow) for workflow and visualization;
- GPU support for Deep Water and NVIDIA;
- Fast training, memory-efficient DataFrame manipulation;
- Easy-to-use algorithms and well documented;

**Cons.**

- Lacks the data manipulation capabilities of R and Pandas DataFrames;
- Slow in learning and supports limited model running at a time.

### Keras

The Python-based Keras (https://keras.io/) library is used on top of Theano or TensorFlow. Its models can be imported to DL4J (see section 5.3). It was developed as a user friendly tool enabling fast experimentation, and easy and fast prototyping. Keras supports CNN, GAN, RNN, and DBN [252].

**Pros.**

- Rich documentation;
- A high-level API for neural networks;
- Ability to run on top of state-of-the-art deep learning libraries/frameworks such as TensorFlow, CNTK, or Theano.

**Cons.**

- Cannot utilize multi-GPU directly;
- Requires Theano as backend for OpenMP support and Theano/TensorFlow/PlaidML as backend for OpenCL.

### Lasagne

Lasagne (http://lasagne.readthedocs.io) DL library is built on top of Theano. It allows multiple input, output, and auxiliary classifiers. It supports user-defined cost functions and provides many optimization functions. Lasagne supports CNN, GAN, RNN, and LSTM.

**Pros.**

- Lasagne is a lightweight library to build and train DL algorithms in Theano;
- Layers, regularizers, and optimizers can be used independently;
- Clear documentation is available;
- Supports training the network on a GPU.

**Cons.**

- Small community than TensorFlow.

### Microsoft Cognitive Toolkit

Replacing CNTK, the Microsoft Cognitive Toolkit (MCT, https://cntk.ai/) is mainly coded in C++. It provides implementations of various learning rules and supports different DL architectures including DNN, CNN, RNN, and LSTM.

**Pros.**

- It is a framework for feedforward DNNs, CNN and RNN;
- Can train production systems very fast;
- Can achieve state-of-the-art performance on benchmark tasks;
- Allow directed graph visualization.

**Cons.**

- Less community support;
- Difficult to install;
- Draw lass interest among the research community.

### MXNet

MXNet (https://mxnet.io/) framework allows defining, training, and deploying deep NN (DA, CNN, GAN, RNN and LSTM) on a wide range of devices—from cloud infrastructure to mobile or even embedded devices (e.g. Raspberry Pi). Written in C++, it is memory efficient and supports Go, JavaScript, Julia, MATLAB, Perl, Python, R, and Scala.

**Pros.**

- A DL framework which has a high-performance imperative API;
- Rich Language support;
- MXNet features advanced GPU support;
- Highly scalable.

**Cons.**

- Small community than TensorFlow;
- Poor API documentation;
- Less popular with the research community.

### Neon

Neon (www.nervanasys.com/technology/neon/) is a DL framework written in Python. It provides implementations of various learning rules, along with functions for optimization and activation. Its support for DL architecture includes CNN, GAN, RNN, LSTM, and DA.

**Pros.**

- Better visualization properties than other frameworks;
- Apply optimization at data loading level,

**Cons.**

- Small community than TensorFlow;
- Less popular with the research community.

### PyTorch

PyTorch (http://pytorch.org/) provides Torch modules in Python. More than a wrapper, its deep integration allows exploiting the powerful features of Python. Inspired by Chainer, it allows dynamic network creation for variable workload and supports CNN, GAN, RNN and LSTM.

**Pros.**

- Pretrained models are available;
- OpenCL support via separately maintained package.
- Easily combine modular pieces;
- Easy to create a layer and run on GPU.

**Cons.**

- Requires writing training code;
- Limited documentation.

### Singa

Singa (https://singa.incubator.apache.org/), it is a distributed DL platform written in C++, Java, and Python.

Its flexible architecture allows synchronous, asynchronous, and hybrid training frameworks to run. It supports a wide range of DL architectures including CNN, RNN, RBM, and DBM.

**Pros.**

- Pretrained models are available;
- Supports model/data or hybrid partitioning, and synchronous/asynchronous/hybrid training;
- Distributed deep learning system and handle Big data.
- Widely used for healthcare data analytics.

**Cons.**

- No Open Multi-Processing support.

### TensorFlow

TensorFlow (www.tensorflow.org), written in C++ and Python, was developed by Google and supports very large-scale deep NN. Amended recently as ‘TensorFlow Fold’, its capability to dynamically create graphs made the architecture flexible, allowing deployment to a wide range of devices (e.g., multi-CPU/GPU desktop, server, mobile devices, etc.) without code rewriting [253, 254]. Also it contains a data visualization tool named TensorBoard and supports many DL architectures including CNN, GAN, RNN, LSTM, and RBMs [255].

**Pros.**

- Handles large-scale data and operate in heterogeneous environments;
- Faster compile time than Theano;
- Computational graph abstraction;
- Supports parallelism.
- TensorBoard is used for workflow and visualization.

**Cons.**

- Large memory footprint;
- Less number of pretrained models are available;
- Computational graph can be slow;
- No support for matrix operations;
- Difficulties in debugging.

### TF.Learn

TF.Learn (www.tflearn.org) is a TensorFlow (see section 5.13)-based high-level Python API. It supports fast prototyping with modular NN layers and multiple optimizers, inputs, and outputs. Supported DL architectures include CNN, GAN, BRNN, and LSTM.

**Pros.**

- Modular and transparent DL library built on the top of TensorFlow;
- Provides a higher-level API to TensorFlow.

**Cons.**

- Slower compared to its competitors.

### Theano

Theano (www.deeplearning.net/software/theano/) is a Python library that builds on core packages like NumPy and SymPy. It defines, optimizes, and evaluates mathematical expressions with tensors and served as foundation for many DL libraries.

**Pros.**

- High flexibility;
- High computational stability;
- Well suited for tensor-based mathematical expressions;
- Open-source libraries such as Keras, Lasagne and Blocks built on the top of Theano;
- Able to visualize convolutional filters, images, and graphs;
- High-level wrappers like Keras and Lasagne increases usability.

**Cons.**

- Difficult to learn;
- Difficult to deploy;
- Deployed on single GPU;
- Slower compilation time than TensorFlow.

### Torch

Started in 2000, Torch (http://torch.ch/), a ML library and scientific computing framework, has evolved as a powerful DL library. Core functions are implemented in C and the rest via LuaJIT scripting language made Torch superfast. Software giants like Facebook and Google use Torch extensively. Recently, Facebook’s DL modules (fbcunn) focusing on CNN have been open-sourced as a plug-in to Torch.

**Pros.**

- User friendly;
- Convenient for employ with GPUs;
- Pretrained models are available;
- Highly modular;
- Easy to create a layer and run on GPU.

**Cons.**

- Special data format and requires conversion;
- Require to write training code;
- Less documentation available.

**Fig. 4:**
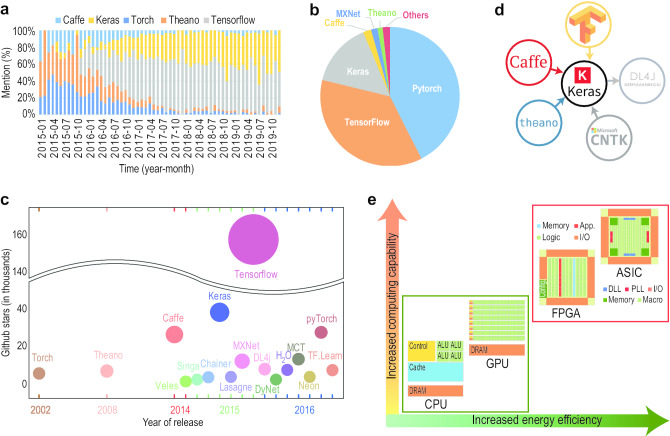
Relative comparison of DL tools. **a** Popularity trend of individual DL tools as per mention in google search generated globally (data courtesy: Google Trend). **b** Mention in articles submitted to arXiv preprint server during the first quarter of 2020. **c** The effect of community’s participation on individual tools is shown by the bubble size, which is product of normalized number of GitHub forks and contributors. **d** As for the interoperability among the DL tools, Keras allows model importing from Caffe, MCT (CNTK), Theano, and TensorFlow and lets DL4j to import. **e** Regarding hardware-based scalability of the DL tools, most of the tools provide CPU and GPU support, whereas FPGA and ASIC can mainly execute pretrained models

### Veles

Veles (https://github.com/Samsung/veles) is a Python-based distributed platform for rapid DL application development. It provides machine learning and data processing services and supports IPython notebooks. Developed by Samsung, one of its advantages is that it supports OpenCL for cross-platform parallel programming, and allows execution across heterogenous platforms (e.g. servers, PC, mobile, and embedded devices). The supported DL architectures include DA, CNN, RNN, LSTM, and RBM.

**Pros.**

- Distributed platform support;
- Supports Jupyter Notebook;
- Supports OpenCL for cross-platform parallel programming.

**Cons.**

- Less community support;
- Draws lass interest from the research community.

## Relative Comparison of DL Tools

To perform relative comparison among the available open-source DL tools, we selected four metrics which are detailed below: trend in their usage, community participation in their development, interoperability among themselves, and their scalability (Fig. 4).

### Trend

To assess the popularity and trend of the various DL tools among the DL consumers, we looked into two different sources to assess the utilization of the tools. Firstly, we extracted globally generated search data from Google Trends^1^ for five years (January 2015 to December 2019) related to search terms consisting of $\langle [tool name] + Deep Learning\rangle .$ The data showed a progressive increase of search about TensorFlow since its release followed by Keras (Fig. 4a). Secondly, mining the content of around 2,000 papers submitted to arXiv’s cs.[CV | CL | LG | AI | NE], and stat.ML categories, during the first quarter of 2020 (i.e. January to March), for the presence of the tool names [256]. As seen in Fig. 4b which shows the percentage of each individual tool’s mention in the papers, the top six tools were identified as: PyTorch, TensorFlow, Keras, Caffe, MXNet, and Theano.

### Community

The community-based development score for each tool discussed in Section 5 was calculated from repository popularity parameters of GitHub (https://github.com/) (i.e., star, fork, and contributors). The bubble plot shown in Fig. 4c depicts community involvement in the development of the tools indicating the year of initial stable release. Each bubble size in the figure, pertaining to a tool, represents the normalized combined effect of fork and contributors of that tool. It is clearly seen that a very large part of the community effort is concentrated on TensorFlow, followed by Keras and Caffe.

### Interoperability

In today’s cross-platform development environments, an important measure to judge a tool’s flexibility is its interoperability with other tools. In this respect, Keras is the most flexible one whose high-level neural networks are capable of running on top of either Tensor or Theano. Alternatively, DL4j model imports neural network models originally configured and trained using Keras that provides abstraction layers on top of TensorFlow, Theano, Caffe, and CNTK backends (Fig. 4d).

### Scalability

Hardware-based scalability is an important feature of the individual tools (Fig. 4e). Today’s hardware for computing devices are dominated by graphics processing units (GPUs) and central processing units (CPUs). But considering increased computing capacity and energy efficiency, the coming years are expected to witness expanded role for other chipset types including application-specific integrated circuits (ASICs) and field-programmable gate arrays (FPGAs). So far DL has been predominantly used through software. The requirement for hardware acceleration, energy efficiency, and higher performance has driven the development of chipset-based DL systems.

**Fig. 5:**
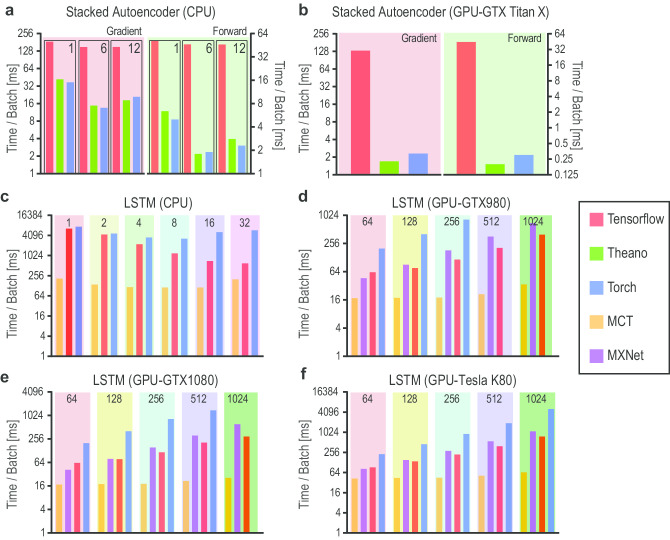
Benchmarking stacked autoencoder or DA **(a, b)** and LSTM **(c-f)** in CPU and GPU platforms. The numbers in **(a, c)** denote the number of CPU threads employed in the benchmarking process, and in **(d-f)** denote the batch size. In case of DA the batch size was 64

## Performance of Tools and Benchmark

The power of DL methods lies in their capability to recognize patterns for which they are trained. Despite the availability of several accelerating hardware (e.g., multicore [C/G]PUs/FPGAs), this training phase is very time-consuming, cumbersome, and computationally challenging. Moreover, as each tool provides implementations of several DL architectures and often emphasizing separate components of them on different hardware platforms, selecting an appropriate tool suitable for an application is getting increasingly difficult. Besides, different DL tools have different targets, e.g., Caffe targets applications, whereas Torch and Theano are more for DL research. To facilitate scientists in picking the right tool for their application, scientists benchmarked the performances of the popular tools concerning their training times [257, 258]. Moreover, to the best of our knowledge, there exist two main efforts that provide the benchmarking details of the various DL tools and frameworks publicly [259, 260]. Summarizing those seminal works, below we provide the time required to complete the training process as a performance measure of four different DL architectures (e.g., FCN, CNN, RNN, and DA) among the popular tools (e.g., Caffe, CNTK, MXNET, Theano, TensorFlow, and Torch) on multicore [C/G]PU platforms.

**Table 8:** Hardware configuration of the evaluating setup

ESN	Processor	Memory
1	**CPU:** E5-1650^a^ @ 3.50 GHz	32 GB
	**GPU:** Nvidia GeForce GTX Titan X^b^	
2	**CPU:** E5-2630^c^ @ 2.20 GHz	128 GB
	**GPU:** Nvidia GeForce GTX 980^d^	
	**GPU:** Nvidia GeForce GTX 1080^e^	
	**GPU:** Tesla K80 accelerator with GK210 GPUs^f^	
3	**CPU:** E5-2690^c^ @ 2.60 GHz	256 GB
	**GPU:** Tesla P100 accelerator^g^	
	**GPU:** Tesla M40 accelerator^h^	
	**GPU:** Tesla K80 accelerator with GK210 GPUs^f^	

*ESN* Experimental Setup Numbers

^a^Intel Xeon CPU v2

^b^3072 cores, 1000 MHz base clock, 12 GB memory

^c^Intel Xeon CPU v4

^d^2048 cores, 1126 MHz base clock, 4 GB memory

^e^2560 cores, 1607 MHz base clock, 8 GB memory

^f^Tesla K80 accelerator has two Tesla GK210 GPUs with 2496 cores, 560 MHz base clock, 12 GB memory

^g^3584 cores, 1189 MHz base clock, 16 GB memory

^h^3072 cores, 948 MHz base clock, 12 GB memory

Table 8 lists the experimental setups used in benchmarking the specified tools. Mainly three different setups, each with Intel Xeon E5 CPU, were utilized during the process. Though the CPU was similar, the GPU hardware was different: GeForce GTX Titan X, GTX 980, GTX 1080, Tesla K80, M40, and P100.

**Fig. 6:**
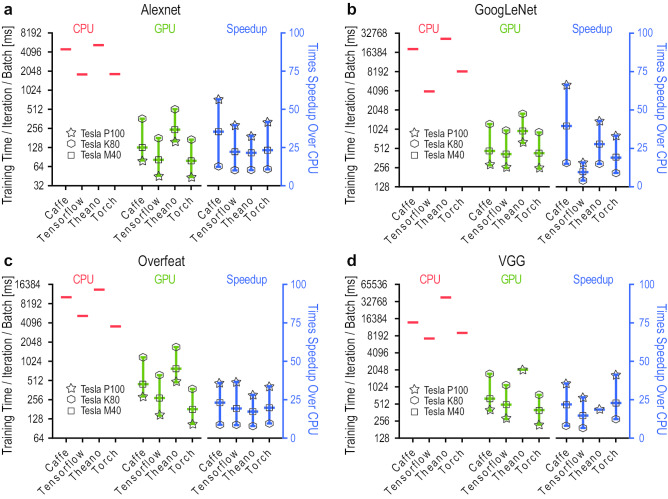
The speedup of CNN training in different DL tools across various GPUs in comparison to CPU. The reported values were calculated for a batch size of 128, except for VGG for which the batch size was 64

Stacked autoencoders or DA were benchmarked using the experimental setup number 1 in Table 8. To estimate the performance of the various tools on implementing DA, three autoencoders (number of hidden layers: 400, 200, and 100, respectively) were stacked with tied weights and sigmoid activation functions. A two-step network training was performed on the MNIST dataset [261]. As reported in Fig. 5 (a, b), the performances of various DL tools are evaluated using forward runtime and training time. The forward runtime refers to the required time for evaluating the information flow through the full network to produce the intended output for an input batch, dataset, and network. In contrast, the gradient computation time measures the time that required to train DL tools. The results suggest that, regardless of the number of CPU threads used or GPU, Theano and Torch outperform TensorFlow in both gradient and forward times (Fig. 5 a, b).

Experimental setup number 2 (Table 8) was used in benchmarking RNN. The adapted LSTM network [262] was designed with 10000 input and output units with two layers and $\sim$13 millions parameters. As the performance of RNN depends on the input length, an input length of 32 was used for the experiment. As the results indicate (Fig. 5 c-f), MCT outperforms other tools on both CPU and all three GPU platforms. On CPUs, TensorFlow performs little better than Torch (Fig. 5 c). On GPUs, Torch is the slowest with TensorFlow and MXNet performing similarly (Fig. 5 d-f).

Still a large portion of the pattern analysis is done using CNN; therefore, we further focused on CNN and investigated how the leading tools performed and scaled in training different CNN networks in different GPU platforms. Time speedup of GPU over CPU is considered as a metric for this purpose. The individual values are calculated using the benchmark scripts of DeepMark [259] on experimental setup number 3 (Table 8) for one training iteration per batch. The time needed to execute a training iteration per batch equals the time taken to complete a forward propagation operation followed by a backpropagation operation. Figure 6 summarizes the training time per iteration per batch for both CPU and GPUs (left y-axis) and the corresponding GPU speedup over CPU (right y-axis).

These findings for four different CNN network models (i.e. Alexnet [92], GoogLeNet [94], Overfeat [263], and VGG [93]) available in four tools (i.e. Caffe, TensorFlow, Theano, and Torch) [264] clearly suggest that network training process is much accelerated in GPUs in comparison to CPUs. Moreover, another important message is that, all GPUs are not the same and all tools don’t scale up at the same rate. The time required to train a neural network strongly depends on which DL framework is being used. As for the hardware platform, the Tesla P100 accelerator provides the best speedup with Tesla M40 being the second and Tesla K80 being the last among the three. In CPUs, TensorFlow achieves the least training time indicating a quicker training of the network. In GPUs, Caffe usually provides the best speedup over CPU but TensorFlow and Torch perform faster training than Caffe. Though TensorFlow and Torch have similar performances (indicated by the height of the lines), Torch slightly outperforming TensorFlow in most of the networks. Finally, most of the tools outperform Theano.

## Open Issues and Future Perspectives

The brain has the capability to recognize and understand patterns almost instantaneously. Over several decades, scientists have been trying decode the biological mechanism of natural pattern recognition that takes place in the brain and translate those principles into AI systems. The increasing knowledge about the brain’s information processing policies enabled this analogy to be adopted and implemented in computing systems. Recent technological breakthroughs, seamless integration of diverse techniques, better understanding of the learning systems, declination of computing costs, and expansion of computational power empowered computing systems to reach human-level computation in certain scenarios [265]. Nonetheless, many of these methods require improvements. Though admittedly, there are distinctions on how a DL-based method can be used and applied on biological data, however, the common open issues and challenges are equally applicable and important for biological data. We identify below shortcomings and bottlenecks of the popular methods, open research questions, and challenges and outline possible directions which requires attention in the near future.

First of all, DL methods usually require large datasets. Though the computing cost is declining with increasing computational power and speed, it is not worthwhile to apply DL methods in cases of small to moderate sized datasets. This is particularly so as considering that many of the DL methods perform continuous geometric transformations of one data manifold to another with an assumption that there exist learnable transfer functions which can perform the mapping [266]. However, in cases when the relationships among the data are causal or very complex to be learned by the geometric transformations, the DL methods fail regardless the size of the dataset [267]. Also, interpreting high-level outcomes of DL methods is difficult due to inadequate in-depth understanding of the DL theories which causes many of such models to be considered as ‘Black box’ [268]. Moreover, like many other ML techniques, DL is also susceptible to misclassification [269] and overclassification [270].

Additionally, the ability to exploit the full benefits offered by open access data repositories, in terms of data sharing and reuse, is often hampered by the lack of unified reporting data standards and non-uniformity of reported information [271]. Data provenance, curation, and annotation of these biological big data are huge challenges too [272].

Furthermore, except for very few large enterprises, the power of distributed and parallel computation through cloud computing remains largely unexplored for the DL techniques. Due to the fact that the DL techniques require retraining for different datasets, repeated training becomes a bottleneck for cloud computing environments. Also, in such distributed environments, data privacy and security concerns are still prevailing [273], and real-time processing capability of experimental data is underdeveloped [274].

To mitigate the shortcomings and address the open issues, the existing theoretical foundations of the DL methods need to be improved. The DL models are required not only to be able to describe specific data but also generalize them on the basis of experimental data which is crucial to quantify the performances of individual NN models [275]. These improvements should take place in several directions and address issues like quantitative assessment of individual model’s learning efficiency and associated computational complexity in relation to well-defined parameter tuning strategies, the ability to generalize and topologically self-organize based on data-driven properties. Also, to facilitate intuitive and less cumbersome interpretation of the analysis results, novel tools for data visualization should be incorporated in the DL frameworks.

Recent developments in combined methods pertaining to deep reinforcement learning (deep RL) have been popularly applied to many application domains (for a review on deep RL, see [276]). However, deep RL methods have not yet been applied to biological pattern recognition problems. For example, analysing and aggregating dynamically changing patterns in biological data coming from multiple levels could help to remove data redundancy and discover novel biomarkers for disease detection and prevention. Also, novel deep RL methods are needed to reduce the currently required large set of labelled training data.

Renewing efforts are required for standardization, annotation, curation, and provenance of data and their sources along with ensuring uniformity of information among the different repositories. Additionally, to keep up with the rapidly growing big data, powerful and secure computational infrastructures in terms of distributed, cloud, and parallel computing tailored to such well-understood learning mechanisms are badly needed. Lastly, there are many other popular DL tools (e.g., Keras, Chainer, Lasagne) and architectures (e.g., DBN) which need to be benchmarked providing the users with a more comprehensive list to choose. Also, the currently available benchmarks are mostly performed on non-biological data, and their scalability to biological data is poor; thus, specialized benchmarking on biological data are needed.

In order to derive insights from an image, a sequence or a signal analysis problem, a selected DL algorithm using a library or a tool (e.g., TensorFlow, Keras, PyTorch, etc.) may need to integrate with a big data framework (e.g., Hadoop, Spark, etc.). In such cases, troubleshooting in the model and debugging the code may be very challenging for the system designer due to the parallel execution of multiple threads which may not always execute in an orderly fashion. The lack of documentation and model transparency of these libraries may make it impossible for the project manager to estimate efforts required in successful completion of a project.

## Conclusion

The diverse biological data coming from different application domains are multimodal, multidimensional, and complex in nature. At present, a huge amount of such data is publicly available. The affordable access to these data came with a huge challenge to analyse and recognize patterns in them which require sophisticated ML tools to do the job. As a result, many ML-based analytical tools have been developed and reported over the last decades and this process has been facilitated greatly by the decrease of computational costs, increase of computing power, and availability of cheap storage. With the help of these learning techniques, machines have been trained to understand and decipher complex patterns and interactions of variables in biological data. To facilitate a wider dissemination of DL techniques applied to biological data and serve as a reference point, this article provides a comprehensive survey of the literature on those techniques’ application on biological data and the relevant open-access data repositories. It also lists existing open-source tools and frameworks implementing various DL methods and compares these tools for their popularity and performance. Finally, it concludes by pointing out some open issues and proposing some future perspectives.

## Footnotes

1 https://trends.google.com
